# Linking cytochrome P450 enzymes from *Mycobacterium tuberculosis* to their cognate ferredoxin partners

**DOI:** 10.1007/s00253-018-9299-4

**Published:** 2018-08-22

**Authors:** Sandra Ortega Ugalde, Coen P. de Koning, Kerstin Wallraven, Ben Bruyneel, Nico P. E. Vermeulen, Tom N. Grossmann, Wilbert Bitter, Jan N. M. Commandeur, J. Chris Vos

**Affiliations:** 1 Division of Molecular Toxicology, Amsterdam Institute for Molecules Medicines and Systems (AIMMS), Faculty of Sciences, Vrije Universiteit, De Boelelaan 1108, 1081 HZ Amsterdam, The Netherlands; 2 Division of Organic and Peptide Chemistry, Vrije Universiteit, Amsterdam, The Netherlands; 3 Division of Analytical Chemistry, Vrije Universiteit, Amsterdam, The Netherlands; 4 Division of Molecular Microbiology, Faculty of Sciences, Vrije Universiteit, Amsterdam, The Netherlands

**Keywords:** Cytochrome P450, Ferredoxin, *Mycobacterium tuberculosis*, NAD (P) H ferredoxin reductase, Redox partners

## Abstract

*Mycobacterium tuberculosis* (*Mtb*) codes for 20 cytochrome P450 enzymes (CYPs), considered potential drug-targets due to their essential roles in bacterial viability and host infection. Catalytic activity of mycobacterial CYPs is dependent on electron transfer from a NAD (P)H-ferredoxin-reductase (FNR) and a ferredoxin (Fd). Two FNRs (FdrA and FprA) and five ferredoxins (Fdx, FdxA, FdxC, FdxD, and Rv1786) have been found in the *Mtb* genome. However, as of yet, the cognate redox partnerships have not been fully established. This is confounded by the fact that heterologous redox partners are routinely used to reconstitute Mtb CYP metabolism. To this end, this study aimed to biochemically characterize and identify cognate redox partnerships for Mtb CYPs. Interestingly, all combinations of FNRs and ferredoxins were active in the reduction of oxidized cytochrome c, but steady-state kinetic assays revealed FdxD as the most efficient redox partner for FdrA, whereas Fdx coupled preferably with FprA. CYP121A1, CYP124A1, CYP125A1, and CYP142A1 metabolism with the cognate redox partners was reconstituted in vitro showing an unanticipated selectivity in the requirement for electron transfer partnership, which did not necessarily correlate with proximity in the genome. This is the first description of microbial P450 metabolism in which multiple ferredoxins are functionally linked to multiple CYPs.

**Electronic supplementary material:**

The online version of this article (10.1007/s00253-018-9299-4) contains supplementary material, which is available to authorized users.

## Introduction

*Mycobacterium tuberculosis* (Mtb), the etiologic agent of tuberculosis (TB), infects approximately one third of the world’s population and it is expected that 5–10% of this population will develop active TB at some point of their lifetime. The global threat to human health caused by TB has been exacerbated in the recent years due to the spread of multi-, extensive-, and total-drug-resistant Mtb strains (Davies 2003; Frieden et al. 2003). Mtb possesses 20 cytochrome P450 enzymes (CYPs), which catalyze stereo- and regio-selective oxidation of endogenous precursors, such as lipids. Although for several CYPs a substrate has not been identified yet, the importance of several CYPs in Mtb pathogenicity and viability has highlighted their potential use as novel drug targets (Sassetti and Rubin 2003). The activity of CYPs is dependent on associated redox partners that transfer the electrons from the cofactor NAD (P) H to the CYP heme center (McLean et al. 2005). CYPs can be classified based on the type of redox systems, with all mycobacterial CYPs belonging to class I, whose catalytic activity is dependent on a NAD (P)H-ferredoxin-reductase (FNR) and a ferredoxin (Fd). In this case, the electron transfer occurs in the following order: NAD (P) H → FNR → Fd → CYP. FNRs generally contain flavin-adenine-dinucleotide (FAD) as cofactor which is able to transfer the electrons from NAD (P) H to ferredoxin. Ferredoxins are low-molecular-weight, acidic and soluble iron-sulfur proteins. The iron-sulfur clusters in ferredoxins can be classified in various types, namely [2Fe-2S], [3Fe-4S], and [4Fe-4S] (Meyer 2008).

**Fig. 1:**
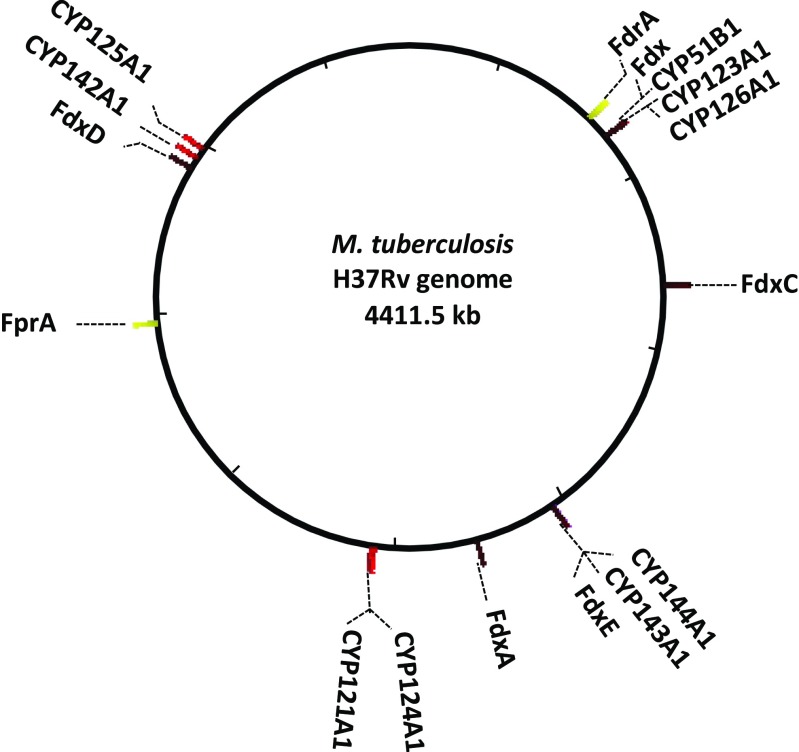
Visual representation of the location of FdrA (*Rv0688*), FprA (*Rv3106*), Fdx (*Rv0763c*), FdxA (*Rv2007c*), FdxC (*Rv1177*), FdxD (*Rv3503c*), FdxE (*Rv1786*), CYP121A1 (*Rv2276*), CYP123A1 (*Rv0766c*), CYP124A1 (*Rv2266*), CYP125A1 (*Rv3545c*), CYP126A1 (*Rv0778*), CYP142A (*Rv3518c*), CYP143A1 (*Rv1785c*), and CYP144A1 (*Rv1777*) in the *Mtb* H37Rv genome. Genomic location of the depicted genes was retrieved from Institut Pasteur GenoList World-Wide

Analysis of *Mtb*’s genome indicated the coding potential for five ferredoxins, Fdx (*Rv0763c*), FdxA (*Rv2007c*), FdxC (*Rv1177*), FdxD (*Rv3503c*), and Rv1786; two FNRs namely FdrA (*Rv0688*) and FprA (*Rv3106*); and two Fd-FNR fusions, FdxB (*Rv3554*) and FprB (*Rv0886*), which could be electron transfer partners for CYPs. The crystal structure for FprA revealed the key amino acids involved in either interaction with NADP^+^ or shielding of the FAD cofactor. It provided crucial information in understanding the molecular interactions with the cognate electron transfer partners (Fischer et al. 2002). The ability of the FprA-Fdx redox system to reduce CYP51B1 (*Rv0764c*) was shown in vitro (McLean et al. 2006). FdrA was also shown to be capable of supporting CYP51B1 activity through the same electron transfer system, thus establishing the first full electron transfer chain for a Mtb CYP (Zanno et al. 2005). Although CYP121A1 was reduced by a Mtb class I redox system formed by FprA and Fdx, conversion of the endogenous substrate was not reported (McLean et al. 2008). Recently, Lu et al. characterized ferredoxin Rv1786 which is located in close proximity to CYP143A1 and CYP144A1 in the *Mtb* H37Rv genome (Fig. 1). Rv1786 displays a tight binding to CYP143A1 (*K*_*D*_ value of 3.37 × 10^−7^ M) (Lu et al. 2017). As yet, the lack of physiological substrates for CYP143A1 and CYP144A1 has hampered the confirmation of this potential electron transfer system as a redox partner for these enzymes.

Despite the existence of several putative Mtb CYP redox partners, the uncertainty about functional oxidase and redox partnerships as well as the difficulties in heterologous expressions in *E. coli* have prompted researchers to routinely use non-physiological reductase partners from *E. coli* or spinach (Belin et al. 2009; Johnston et al. 2009; Driscoll et al. 2010; Ouellet et al. 2010a). A detailed picture of the electron transfer partner preferences for specific and essential biosynthetic CYP pathways in Mtb is lacking. To date, the two FNRs (FdrA and FprA) and two out of five ferredoxins (Fdx and Rv1786) have been characterized (Fischer et al. 2002; Zanno et al. 2005; McLean et al. 2006; Lu et al. 2017). Based on their genomic location, most plausible candidate electron transfer partnerships are Fdx-CYP51B1/CYP123A1/CYP126A1, FdxD-CYP125A1/CYP142A1, and FdxE-CYP143A1/CYP144A1 (Fig. 1). From these possible partnership combinations, only CYP51B1 driven 14 α-lanosterol demethylation has been successfully reconstituted with the proteins encoded by the direct gene neighbor Fdx (*Rv0763c*) and the chromosomally distant genes FdrA (*Rv0688*) and FprA (*Rv3106*) (Fig. 1) (Fischer et al. 2002; Zanno et al. 2005; McLean et al. 2006).

In this study, all putative Mtb ferredoxins, except FdxC, were cloned, heterologously expressed in *E. coli*, characterized and combined with several relevant Mtb CYPs. Our results show that CYP121A1, CYP124A1, CYP125A1, and CYP142A1 catalytic activities can only be supported by specific cognate redox partners in vitro, demonstrating that the molecular interactions in electron transfer partners for these Mtb CYPs are highly selective.

## Materials and methods

### Materials, template DNA, and strains

Genomic DNA from Mtb strain H37Rv, which was kindly provided by Prof. Dr. Wilbert Bitter, Medical Microbiology and Infection Control, VU Medical Center, Amsterdam, The Netherlands, was used as template DNA in this study. The Mtb strain H37Rv was deposited at the American Type Culture Collection as ATCC 25618. *E. coli* strains DH5α, BL21 (DE3), and Rosetta (DE3). The *E. coli* strains DH5α and BL21 (DE3) were obtained from TaKaRa (Dalian, China), and the *E. coli* strain Rosetta (DE3) was a kind gift from Prof. Dr. Wilbert Bitter. The pGro7 chaperone plasmid was purchased from TaKaRa (Dalian, China). Cholesterol and TMS derivatization agents were obtained from Sigma (Schnelldorf, Germany). All other chemicals and reagents were of analytical grade and obtained from standard suppliers.

### Sequence similarity analysis

Protein sequences were obtained from the NCBI database. Sequence similarity alignment was conducted using the Multiple Sequence Alignment program Clustal Omega. Percent identity matrix was generated by Clustal 2.1 (Goujon et al. 2010; Sievers et al. 2014).

### Molecular biology

The PCR amplification of the genes coding for the putative proteins described in this study was conducted as published by Ouellet et al. (2008). Amplicons were subcloned into the pET28a(+) vector with primers depicted in Supplemental Table S5, creating genes with an N-terminal tag of 20 amino acids containing a thrombin cleavage site and 6 histidines to enable purification via nickel-agarose affinity chromatography. The positive clones were further transformed into competent *E. coli* BL21(DE3) cells, with the exception of FdxA which was transformed into *E. coli* Rosetta (DE3) cells together with the pGro7 chaperone plasmid. The correct sequences were verified by DNA sequencing (Macrogen, Amsterdam, the Netherlands).

### Heterologous expression and purification

Transformant *E. coli* BL21 (DE3) and *E. coli* Rosetta (DE3), together with the pGro7 chaperone plasmid for FdxA (*Rv2007c*), containing the recombinant Fdx (*Rv0763c*), FdxA (*Rv2007c*), FdxC (*Rv1177*), FdxD (*Rv3503c*), and FdxE (*Rv1786*), were grown in 15 mL overnight in Luria Bertani broth with kanamycin (30 μg mL^−1^) selection at 37 °C; additional chloramphenicol (50 μg mL^−1^) was also added for FdxA. Terrific broth supplemented with kanamycin (30 μg mL^−1^) and chloramphenicol (50 μg mL^−1^) for FdxA was inoculated with the overnight cultures. Cells were grown at 37 °C until the OD_600_ reached 0.6–0.8. Protein expression was induced by addition of 0.6 mM isopropyl β-d-1-thiogalactopyranoside (IPTG). In addition, 50 μM L cysteine, 50 μM Fe_2_SO_4_, and 50 μM FeCl_3_ were also added to aid in the correct folding of the iron-sulfur cluster. The expression was allowed to proceed for an additional 24 h at 24 °C. Cells were harvested and the pellet was re-suspended in 100 mM potassium phosphate buffer supplemented with glycerol (Kpi, pH 7.4, 10% glycerol, 0.25 mM EDTA, and 0.1 mM DTT). Cells were disrupted by Emulsifex-C3 (Avestin Inc., Ottawa, Ontorio, Canada; 1000 psi. 3 repeats), and the cytosolic fraction was separated from the membrane by ultracentrifugation of the lysate (120,000×*g*, 4 °C, 1.15 h). The soluble extract was used for extraction and protein purification. All proteins were further purified using nickel affinity column as described previously (Damsten et al. 2008) with a yield > 60%. The heterologous expression and purification of FdrA, FprA, CYP121A1, CYP124A1, CYP125A1, and CYP142A1 was conducted as described for ferredoxins with the exception that after FprA and FdrA expression, induction cells were allowed growth at 22 and 25 °C, respectively. The expression of CYP121A1, CYP124A1, CYP125A1, and CYP142A1 was induced with 0.6 mM IPTG in the presence of 0.5 mM of the heme precursor δ-aminolevulinic acid (δ-ALA) and 0.25 mM FeCl_3_. The expression was allowed to proceed at 25 °C for CYP124A1 and CYP125A1, whereas 20 °C was used for CYP121A1 and CYP1421A1. CYP concentration was determined using carbon monoxide (CO) difference spectrum assay as described by Omura and Sato (1964).

### MALDI-TOF MS

Samples were purified using ZipTip C4 following manufacturer’s protocol and 1 μL was embedded in the sandwich matrix consisting of α-cyano-4-hydroxycinnamic acid (CHCA), 2,5-dihydroxy benzoic acid (DHB), and sinapinic acid. The sandwich matrix-assisted laser desorption/ionization MALDI-TOF in the liner positive mode.

### Steady-state kinetics

Steady-state kinetics of electron transfer were determined with 0.2 μM FdrA or FprA and varying concentrations of the electron acceptor dichlorophenolindophenol (DCPIP) (0–500 μM) or different concentrations of the cofactors NAD (P) H in 50 mM potassium phosphate buffer (pH 7.4). DCPIP reduction was measured in an Ultrospec 2000 spectrophotometer at 600 nm for a period of 1 min.

### Identification of endogenous electron transfers

Identification of the endogenous electron transfers was conducted using cytochrome c as final electron acceptor. Reactions were conducted with 0.5 μM FdrA or FprA in presence of different molar ratios (1:0, 1:1, 1:2 or 1:4) of ferredoxins, varying concentrations of NAD (P) H (0–200 μM) in 50 mM potassium phosphate buffer (pH 7.4). Reactions were started by the addition of NADH and NADPH for FdrA and FprA, respectively, in presence of 100 μM cytochrome c. Cytochrome c reduction was recorded in a Ultrospec 2000 spectrophotometer at 550 nm for a period of 1 min.

### Synthesis of cyclo (L-Tyr-L-Tyr)

Beginning from 1 equivalent of Fmoc-Tyr (OtBu)-OH, **1** was coupled with 0.9 equivalents of H-Tyr (OtBu)-OMe and **2** using 1.2 equivalents of EDC-HCl and HOBt each as well as 3.2 equivalents of *N*,*N*-diisopropylethylamine (DIPEA). After full conversion, the reaction mixture was quenched using a saturated aqueous solution of NH_4_Cl. The aqueous phase was extracted with ethyl acetate (EtOAc), twice. The combined organic fractions were dried over MgSO_4_ before the solvent was removed under reduced pressure. The pure dipeptide **3** was obtained after purification on silica (2:1 EtOAc:cyclohexane). Subsequent deprotection of the N- and C-terminus was performed using 1.5 equivalents LiOH in THF:H_2_O (7:3) at 0 °C. After 30 min, the reaction mixture was neutralized by adding 2 M HCl. After washing with cyclohexane, the aqueous solution was freeze-dried overnight. The remaining solid was dissolved in formic acid and stirred for 2 h at room temperature before freeze-drying again overnight. The cyclization of dipeptide **5** was performed in *sec*-butanol/toluene (4:1) under heating to reflux for 5 h. After room temperature, the organic phase was washed with water, dried over MgSO_4_, and the solvent was removed under reduced pressure. The remaining oil was purified using Porapak® column chromatography with 20% acetonitrile in H_2_O to yield pure cyclo (L-Tyr-L-Tyr) **6**. Schematic synthetic pathway is depicted in Supplemental Fig. S5.

### Reconstitution of CYP121A1, CYP124A1, CYP125A1, and CYP142A1 activity

CYP121A1 activity using its cognate redox partners was conducted with 5 μM CYP121 (final concentration) pre-incubated with 100 μM cYY (final concentration) in 50 mM potassium phosphate buffer (pH 7.4) supplemented with 5 mM MgCl_2_. FdrA or FprA and Fds, FdxD, or FdxE in a 1:5:10 M ratio were added to the incubation, and the reaction was started by the addition of a NAD (P) H regeneration system (final concentrations of 0.5 mM NAD (P) H, 10 mM glucose 6-phosphate, and 0.4 unit mL^−1^ glucose-6-phosphate dehydrogenase) in a final volume of 100 μL. After 1 h, the reaction was stopped with 5 μL 80% TFA. Proteins were removed by centrifugation (14,041×*g*, 15 min) and analyzed by HPLC equipped with a time-of-flight (TOF) as described below. CYP124A1, CYP125A1, and CYP142A1 ability to metabolize cholesterol when supported by the endogenous redox partners was assessed. Briefly, 0.5 μM CYP (final concentration) was pre-incubated for 10 min with 50 μM cholesterol (final concentration) from a 9% methyl-β-cyclodextrin (MβCD) stock in 50 mM potassium phosphate buffer (pH 7.4) supplemented with 5 mM MgCl_2_. The selected Mtb CYP, FdrA or FprA, and ferredoxins, either Fdx, FdxA, FdxD, or FdxE, were mixed in a 1:1:2, 1:2:5, or 1:5:10 M ratio in a final volume of 500 μL. Reactions were started by addition of NAD (P) H regeneration system and allowed to proceed for 1 h at 37 °C. Reactions were stopped by addition of 1 volume 1 N HCl containing the internal standard. The substrate, product(s), and internal standard were extracted twice with methyl-*tert*-buthyl-ether and converted to into trimethylsilyl (TMS) ethers. Samples were dissolved in CHCl_3_ and analyzed by GC-MS as described below. All reactions were conducted in duplicates, and control incubations without NAD(P)H regeneration system or redox partners were also conducted. For the generation of the NAD(P)H regeneration system, NADH and NADPH were used for FdrA and FprA, respectively.

### Analytical methods

For the detection of cYY and its metabolites, an HPLC equipped with a time-of-flight (TOF) mass detector and diode-array-detector was used. The system consisted of an Agilent 1200 rapid resolution LC system connected to a Luna 5 μm C18 (4.6 × 150 mm) column. The HPLC-column was connected to an Agilent TOF 6230 mass spectrometer with an electrospray ionization source. The system was operating in positive ionization mode with a capillary voltage of 3500 V, 10 L min^−1^ nitrogen drying gas, and 50 psig nitrogen nebulizing gas at 350 °C. The data were analyzed using Agilent Masshunter Qualitative Analysis software. For the separation of the metabolites, a binary gradient running at 0.6 mL min^−1^ was used consisting of eluent A (99.9% H_2_O and 0.1% formic acid) and eluent B (99.9% acetonitrile and 0.1% formic acid). The first 5 min was isocratic at 0% B, followed by a linear increase from 0% B to 99% B in 20 min. Next, the column was eluted isocratically at 99% B for 10 min after which the column was washed by eluting for 5 min with 0% B.

For the detection of cholesterol and its metabolites, a Shimadzu GC-MS-QP2010 Plus equipped with an AOC-20i autoinjector and an AOC-20S autosampler was used. Samples were separated on a ZB-1 GC-column (30 m × 0.25 mm, 0.10 μm; Phenomenex, Amstelveen, The Netherlands). Ionization was done by electron impact (EI) at 70 eV. Helium (99.999%) was used as carrier gas with a gas flow of 1 mL min^−1^. The GC-MS interface temperature was set to 320 °C, the ion source to 240 °C. The injector temperature was maintained at 270 °C. The temperature program for the GC was as follows: 1 min stationary at 200 °C, ramped up at 20 °C min^−1^ to 280 °C, ramped up at 3 °C min^−1^ to 310 °C and maintained at 310 °C for 5 min.

### Statistics

Data are represented as means of duplicates ± S.D. Each value corresponds to a different incubation conducted separately.

## Results

### Sequence similarity analysis

**Fig. 2:**
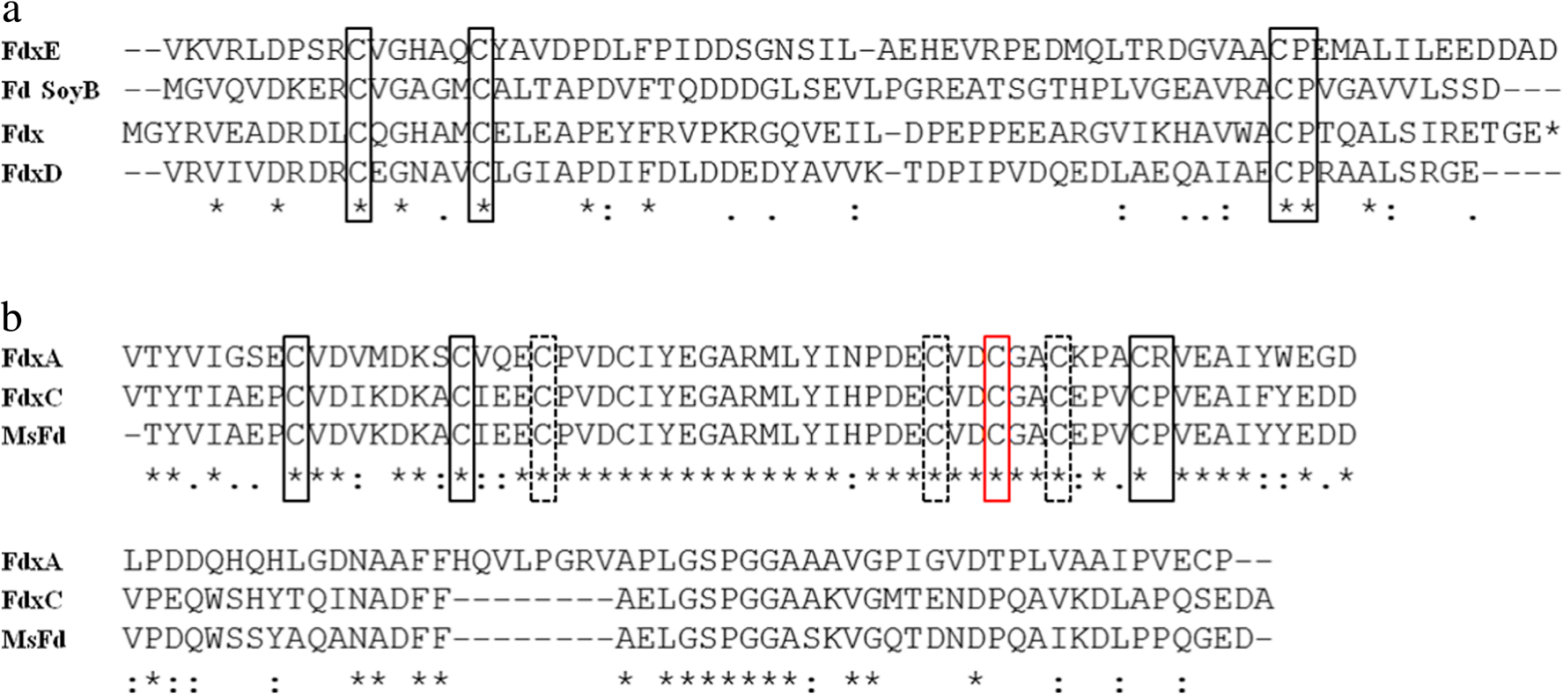
Sequence alignment analysis of ferredoxins from *Mtb* strain H37Rv. **a** Sequence alignment of the [3Fe-4S] ferredoxins, namely Fdx, FdxD, and Rv1786/FdxE and FdsoyB found in *S. griseus*. Cysteines corresponding to the Cys-X_2_-X-X_2_-Cys-X_n_-Cys-Pro iron-sulfur cluster-binding motif are highlighted with a black square. **b** Sequence alignment of the [3Fe-4S] and [4Fe-4S] ferredoxins from Mtb strain H37Rv, namely FdxA and FdxC, and FdxA from *M. smegmatis* (MsFd). Cysteines linked to the [3Fe-4S] Cys-X_2_-X-X_2_-Cys-X_*n*_-Cys-Pro iron-sulfur cluster-binding motif are highlighted with a black square whereas the ones involved in the [4Fe-4S] iron-sulfur cluster-binding motif are highlighted with a dashed black square. The characteristic second Cys found in the Cys-X_2_-X-X_2_-Cys cluster-binding motif in [4Fe-4S] ferredoxins is highlighted with a red square

Ferredoxins contain distinct iron-sulfur clusters, namely [2Fe-2S], [3Fe-4S], and/or [4Fe-4S]. The 3Fe-4S ferredoxins share a common Cys-X_2_-X-X_2_-Cys-X_*n*_-Cys-Pro iron-sulfur cluster-binding motif, where X is any amino acid, and X_*n*_ indicates a number of flexible amino acids (Trower et al. 1993; Green et al. 2003; Sevrioukova 2005). In the [4Fe-4S] containing ferredoxins, the typical binding motif has a fourth cysteine (Cys) in the position underlined. The *Mtb* genome encodes five putative ferredoxins, namely Fdx (*Rv0763c*), FdxA (*Rv2007c*), FdxC (*Rv1177*), FdxD (*Rv3503c*), and *Rv1786*. We believe that it seems appropriate to designate the name FdxE to Rv1786. Analysis of the amino acid sequences of Fdx, FdxD, and FdxE confirmed the presence of the Cys-X_2_-X-X_2_-Cys-X_*n*_-Cys-Pro iron-sulfur cluster-binding motif, consistent with a [3Fe-4S] cluster (Fig. 2a) (Trower et al. 1993; Duff et al. 1996). In contrast, FdxA and FdxC resemble the 7Fe FdxA from *Mycobacterium smegmatis* (*MsFd*), where a [3Fe-4S] and a [4Fe-4S] iron-cluster are combined in a single protein (Fig. 2b) (Ricagno et al. 2007). The conserved terminal Pro in the iron-sulfur cluster-binding motif in FdxA is substituted for an Arg at position 51. FdxA and FdxC have a high sequence identity (58%), and they share 61 and 87% sequence similarity with *MsFd*, respectively. The overall sequence identity between the [3Fe-4S] cluster-containing Mtb Fds; Fdx, FdxD, and FdxE is approximately 30% (Supplemental Table S1).

### Molecular cloning, heterologous expression, and spectroscopic properties

The coding sequences of Mtb FdrA (*Rv06388*), FprA (*Rv3106*), Fdx (*Rv0763c*), FdxA (*Rv2007c*), FdxC (*Rv1177*), FdxD (*Rv3503c*), FdxE (*Rv1786*), CYP121A1 (*Rv2276*), CYP124A1 (*Rv2266*), CYP125A1 (*Rv3545c*), and CYP142A1 (*Rv3518c*) were amplified from *Mtb* H37Rv genomic DNA and cloned into an expression vector encoding an N-terminal 6-His-tag. His-tagging has been used previously to purify ferredoxins, FNRs, and CYPs from several species (e.g., Pandini et al. 2002; Johnston et al. 2009; Pandey et al. 2014; Lu et al. 2017). All proteins were expressed in *E. coli* BL21 (DE3) cells, except for FdxA, which in *E. coli* Rosetta (DE3) was co-expressed with GroE chaperone. Unfortunately, efforts to express FdxC in *E. coli* were not successful.

**Fig. 3:**
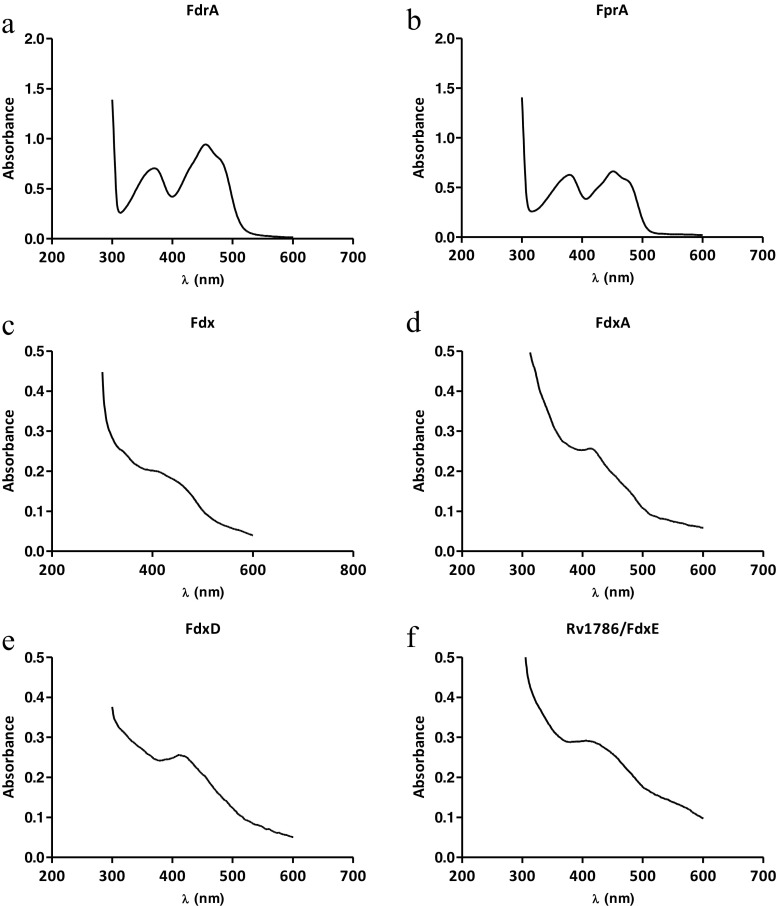
UV/Vis absorption for oxidized forms of purified recombinant proteins. **a** FdrA. **b** FprA. **c** Fdx. **d** FdxA. **e** FdxD. **f** Rv1786. FdrA and FprA showed the characteristic flavin absorbance at between 300 and 500 nm, with bands centered at 380 and 452 nm and shoulders at 422 and 473 nm. Fdx, FdxD, and Rv1786/FdxE showed a broad band at 400 nm with peak at 412 nm, characteristic of [3Fe-4S] Fds whereas FdxA displayed a peak at 425 nm, characteristic of [4Fe-4S] ferredoxins

SDS-PAGE analysis of the purified recombinant proteins showed that CYP121A1, CYP124A1, CYP125A1, CYP142A1, FdrA, FprA, Fdx, and FdxA were > 90% pure with expected apparent molecular masses consistent with the calculated masses of the respective proteins. FdxD and FdxE migrated at 15 and 17 kDa, respectively, about 9–10 kDa larger than the predicted molecular weight. To verify the identity of FdxD and FdxE, MALDI-TOF MS was applied. A major peak at approximately 9 kDa for FdxD and at 9.5 kDa for FdxE was obtained consistent with the calculated molecular weights of the respective proteins (Supplemental Fig. S1). It was previously reported that ferredoxins can migrate with an apparent higher molecular mass from the calculated value (Armengaud et al. 1994; Schiffler et al. 2004; Ewen et al. 2009; Lu et al. 2017). UV/Vis spectroscopic analysis of both FdrA and FprA showed spectra in line with those reported in previous studies displaying the characteristic peaks at 370 and 450 nm (Fig. 3a, b) (Fischer et al. 2002; Zanno et al. 2005). CYP121A1, CYP124A1, and CYP142A1 (5 μM) exhibited typical CYP spectrum with a Soret band at 416–419 nm in the ferric state, while Soret peaks were shifted from around 419 nm in the oxidized form to the characteristic peak at approximately 450 nm in the reduced CO-complex difference forms (dotted line), suggesting a functional heme-domain (Supplemental Fig. S2). In contrast, CYP125A1 showed an unstable P450 spectra, with heme Soret features at 393 nm and a shoulder at 416 nm, where the Fe (II)-CO of complex spectrum has two maxima at 450 (P450) and 422 nm (P420), suggesting protonation of the proximal cysteinate ligand (Cys 377) to a thiol in the P420 form (Supplemental Fig. S2 C). This characteristic Soret feature was also reported by others (McLean et al. 2009; Ouellet et al. 2010a). The relatively featureless UV-spectra for Fdx, FdxD, and FdxE showed only a broad band at ∼ 400 nm with maximum at 412 nm (Fig. 3c, e, f), consistent with the results obtained for the [3Fe-4S] iron-cluster-containing Fdx (McLean et al. 2005), FdxE (Lu et al. 2017), and Fd from *Sulfolobus acidocaldarius* (Duff et al. 1996)*.* In addition, FdxA displayed a band with maximum at 425 nm, consistent with the spectrum reported for other bacterial [4Fe-4S] ferredoxins (Fig. 3d) (Palmer et al. 1967; Golinelli et al. 1998).

### Steady-state kinetics and cofactor preference of Mtb FNRs

The reductase activity of the purified FNRs was investigated using dichlorophenol indophenol (DCPIP) as an electron acceptor. FdrA and FprA were both able to catalyze the electron transfer from the cofactors NADH and NADPH to the artificial electron acceptor DCPIP. Steady-state kinetic studies showed that FdrA was more efficient in DCPIP reduction than FprA, when NADH was used as a cofactor, whereas FprA could reduce DCPIP in the presence of both NADH and NADPH (Supplemental Table S2). Cofactor preference analysis showed that NADH is the preferred cofactor for FdrA (*K*_*m*_ = 92 μM) whereas FprA displayed 71-fold lower *K*_*m*_-value for NADPH than for NADH confirming previously reported results (Fischer et al. 2002; Zanno et al. 2005) (Supplemental Table S2).

### Identification of endogenous electron transfers

The ability of the recombinant proteins Fdx, FdxA, FdxD, and FdxE to reduce cytochrome c was determined in the presence of either FdrA or FprA using their preferred cofactor, NADH and NADPH, respectively, according to established methods (Fischer et al. 2002; Zanno et al. 2005; Lu et al. 2017). The enzyme kinetic constants for the two FNRs coupled with increasing concentrations of ferredoxins in the presence of the substrate cytochrome c are listed in Tables 1 and 2.

**Table 1:** Kinetic parameters for cytochrome c reductase activity of FdrA in the presence of various concentrations of Mtb ferredoxins and using NADH as a cofactor

	Molar ratio	*k*_*cat*_ (min^−1^)	*K*_*m*_^NADH^ (μM)	*k*_*cat*_/*K*_*m*_ (M^−1^ min^−1^) × 10 ^5^	(*k*_*cat*_ /*K*_*m*_)^FdrA:Fd^/(*k*_*cat*_/*K*_*m*_)^FdrA^
FdrA	1:0	0.7 ± 0.1	6.7 ± 2.4	1.1	1
FdrA:Fdx	1:1	2.9 ± 0.1	8.3 ± 1.4	3.5	3.5
	1:2	4.4 ± 0.3	13.4 ± 2.7	3.3	3
	1:4	8.5 ± 0.4	10.8 ± 2.1	7.8	7.5
FdrA:FdxA	1:1	3.5 ± 0.1	3.7 ± 0.6	9.5	9
	1:2	6.7 ± 0.3	5.8 ± 1.2	11	11
	1:4	14.5 ± 0.4	7.8 ± 0.8	19	18
FdrA:FdxD	1:1	28.6 ± 1.9	3.5 ± 1.2	82	78
	1:2	60.4 ± 3.7	5.3 ± 1.4	114	109
	1:4	82.4 ± 5.5	6.4 ± 2.4	130	123
FdrA: FdxE	1:1	10.0 ± 0.6	5.0 ± 0.6	20	20
	1:2	31.4 ± 1.7	4.8 ± 1.1	65	63
	1:4	56.0 ± 1.8	9.3 ± 1.2	60	58

**Table 2:** Kinetic parameters for cytochrome c reductase activity of FprA in the presence of various Mtb ferredoxins using NADPH as a cofactor

	Molar ratio	*k*_*cat*_ (min^−1^)	*K*_*m*_^NADPH^ (μM)	*k*_*cat*_/*K*_*m*_ (M^−1^ min^−1^) × 10 ^5^	(*k*_*cat*_ /K_m_)^FprA:Fd^/(*k*_*cat*_/*K*_*m*_)^FprA^
FprA	1:0	0.6 ± 0.1	4.8 ± 2.3	1.2	1
FprA: Fdx	1:1	19.5 ± 0.4	18.7 ± 1.2	10	8.3
	1:2	36.6 ± 1.4	23.6 ± 2.8	15	12
	1:4	55.2 ± 2.0	16.5 ± 2.5	33	27
FprA:FdxA	1:1	4.8 ± 0.3	18.2 ± 4.5	2.6	2
	1:2	15.4 ± 1.1	47.5 ± 8.9	3.2	2.6
	1:4	30.0 ± 1.2	55.4 ± 5.6	5.4	4.3
FprA:FdxD	1:1	1.1 ± 0.1	1.2 ± 0.4	9.2	7.3
	1:2	1.4 ± 0.1	2.0 ± 0.4	7	5.6
	1:4	2.1 ± 0.1	5.0 ± 1.2	4.2	3.3
FprA:FdxE	1:1	9.0 ± 2.0	14.3 ± 2.8	6.3	5
	1:2	9.5 ± 1.4	10.1 ± 1.2	9.4	7.5
	1:4	14.3 ± 0.9	14.3 ± 3.7	10	8

Both FdrA and FprA showed basal cytochrome c reductase activity in the absence of a ferredoxin as electron transfer partner (*k*_*cat*_ = 0.7 and 0.6 min^−1^, respectively). In the presence of Fdx, catalytic efficiencies (*k*_*cat*_/*K*_*m*_) of cytochrome c reduction were approximately 8- to 27-fold higher for FprA (Table 2) and 3.5- to 7.5-fold higher for FdrA (Table 1). FdxD (78 to 123-fold higher *k*_*cat*_/*K*_*m*_, depending on the molar ratio of the two proteins) proved to be the most efficient partner for FdrA, followed by FdxE (20- to 58-fold higher *k*_*cat*_/*K*_*m*_), FdxA (9- to 18-fold higher *k*_*cat*_/*K*_*m*_), and Fdx (3.5- to 7.5-fold higher *k*_*cat*_/*K*_*m*_) (Table 1). In contrast, Fdx (8- to 27-fold higher *k*_*cat*_/*K*_*m*_) was the most efficient partner for FprA, with minor differences between FdxA (2- to 4-fold higher *k*_*cat*_/*K*_*m*_), FdxD (7- to 3-fold higher *k*_*cat*_/*K*_*m*_), and FdxE (5- to 8-fold higher *k*_*cat*_/*K*_*m*_) (Table 2). An overview of the *k*_*cat*_/*K*_*m*_ derived from this study suggest that specially FdxD, followed by FdxA and FdxE, may interact preferably with the NADH-FdrA system to form an efficient electron transfer chain in vivo whereas Fdx might be the preferred partner for NADPH-FprA. In the absence or presence of FdxD, the *K*_*m*_ values for FdrA and FprA remained relatively unaltered, suggesting that FdxD does not have a significant effect on the affinity of FdrA or FprA for the electron acceptor, cytochrome c.

### Metabolism of specific substrates by Mtb CYPs reconstituted with cognate redox partners

In vitro reconstitution of Mtb CYP121A1, CYP124A1, CYP125A1, and CYP142A1 catalytic activities has previously been conducted using the non-physiological redox partners from either *E. coli* or spinach (Belin et al. 2009; Johnston et al. 2009; Driscoll et al. 2010; Ouellet et al. 2010a). Here, we qualitatively investigated the metabolism of their specific substrates by reconstitution with the putative redox partners from Mtb. Because the optimal stoichiometry of CYP:FNR:Fd is unknown, the molar ratios of the redox partners were varied using *E. coli* redox partners as positive control. In previous studies, successful 1:5:10 and 1:5:20 M ratios CYP:FNR:Fd have been reported (Driscoll et al. 2010; Honda Malca et al. 2012).

**Fig. 4:**
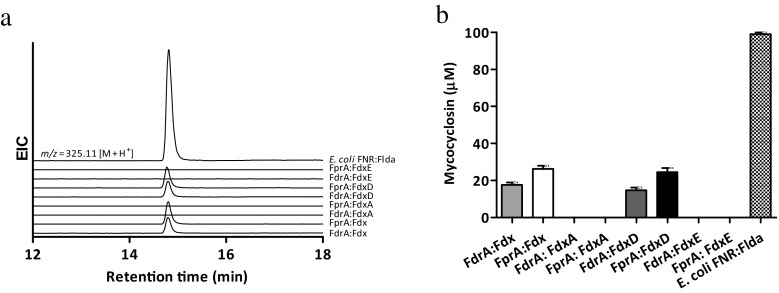
cYY conversion catalyzed by CYP121A1 supported by cognate redox partners. **a** Extracted ion chromatograms (m/z = 325.11 [M + H^+^]) of incubations with cYY (100 μM, final concentration) and CYP121 (5 μM). Incubations were performed in a 1:5:10 M ratio. **b** Mycocyclosin formation by CYP121A1 (5 μM) supported by cognate redox partners. Incubations were conducted in 1:5:10 M ratio. Error bars represent the variability of duplicates

CYP121A1 highly selectively catalyzes the conversion of cyclo (L-Tyr-L-Tyr) (cYY) into mycocyclosin (McLean et al. 2008; Belin et al. 2009). A screening using a 1:5:10 M ratio CYP:FNR:Fd showed that among the four recombinant Mtb ferredoxins used in this study, only Fdx and FdxD were able to support cYY conversion by CYP121A1, independently of the FNR used (Fig. 4a, b). LC-MS analysis of the incubations showed a single product with *m/z* 325.11 ([M + H]^+^), corresponding to a loss of two H-atoms forming mycocyclosin (Fig. 4a). cYY conversion was not seen in the absence of NAD(P)H regeneration system and in the absence of CYP121A1. Incubations conducted using the identified active recombinant redox partners for CYP121A1 showed that both Fdx and FdxD were equally efficient in supporting mycocyclosin formation by CYP121A1, while the overall conversion was slightly higher (≈ 1.5-fold) when FprA was present in the reconstitution system (Fig. 4b). However, the *E. coli* proteins supported a clearly higher conversion of cYY by CYP121A1 (3.7-fold higher), indicating that perhaps the proper endogenous redox partner has not been identified yet. In addition, CYP121A1 has been reported to undergo the peroxide shunt pathway, a reaction by which hydrogen peroxide is generated by the redox partners through reduction of O_2_, resulting in a competition with electron transfer to the CYP enzyme (Dornevil et al. 2017). In order to assess whether the product formation observed for CYP121A1 is derived from the peroxide shunt pathway, the reactions were conducted in the presence of catalase. Our results showed that the peroxide shunt pathway had no significant effect on the turnover of CYP121A1, confirming that mycocyclosin formation resulted from a formal CYP catalytic cycle supported by the cognate redox partners (Supplemental Fig. S3).

**Fig. 5:**
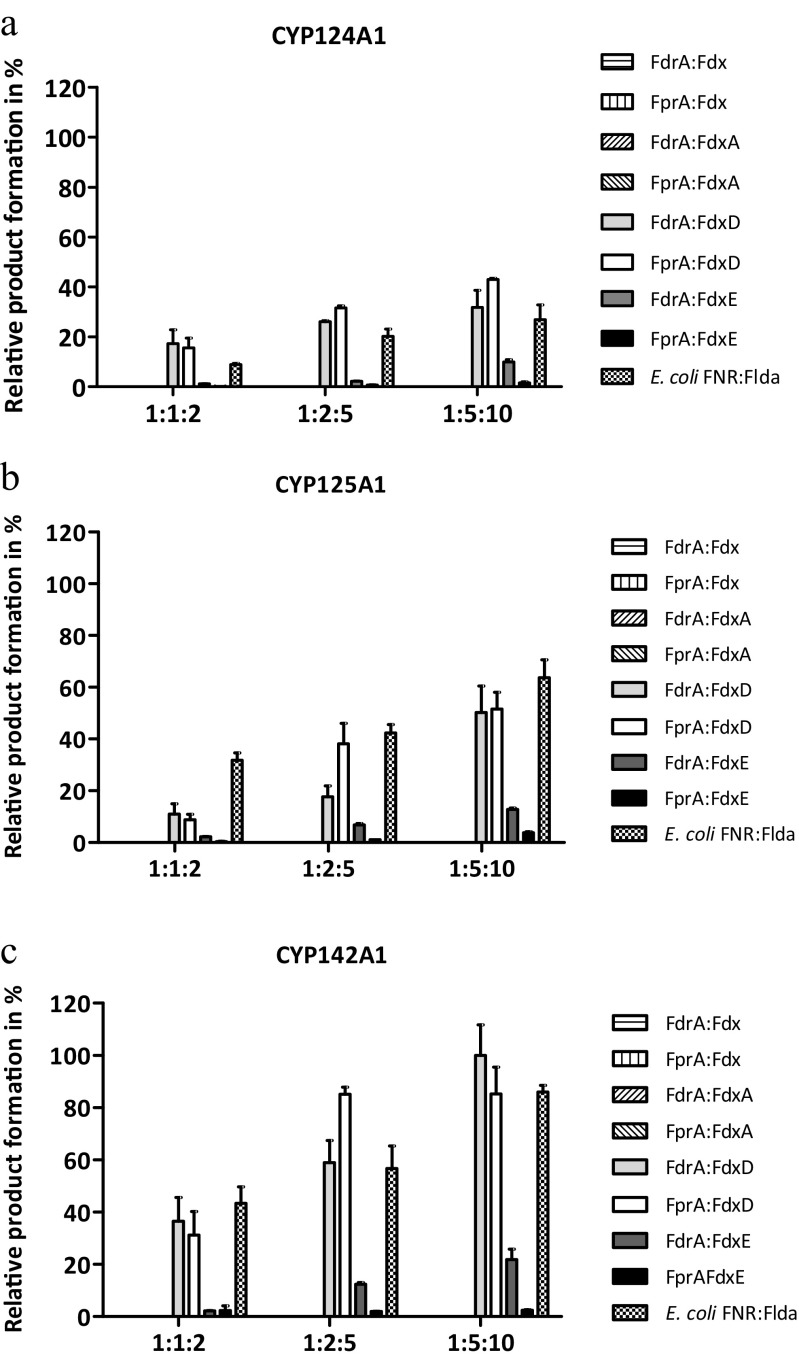
Oxidative metabolism of cholesterol by recombinant Mtb CYPs reconstituted with cognate redox partners. **a** CYP124A1, **b** CYP125A1, and **c** CYP142A1, using different CYP-NAD(P)H FNR:Fd molar ratios. The catalytic system CYP142A1:FdrA:FdxD yielded the higher 27-hydroxycholesterol formation, and therefore, it was at 100% for comparison reasons. Error bars represent the variability of duplicates

Recombinant CYP124A1, CYP125A1, and CYP142A1 catalyze cholesterol conversion, when the electron flow system is reconstituted with surrogate redox partners derived from *E. coli* or spinach (Johnston et al. 2009; Driscoll et al. 2010; Ouellet et al. 2010a). In vitro reconstitution of cholesterol metabolism using Mtb CYPs in combination with the recombinant endogenous redox partners used in this study at different reaction molar ratios (1:1:5, 1:2:5, and 1:5:10) showed that only FdxD and FdxE were able to support cholesterol conversion (Fig. 5) in presence of either of the endogenous FNRs, whereas neither Fdx nor FdxA was able to support these biocatalyst’s catalytic activity. Analysis of the turnover rates revealed that CYP124A1, CYP125A1, and CYP142A1 displayed approximately 4-fold higher product formation, when FdxD over FdxE was used (Supplemental Table S4). CYP142A1 was found to be the most efficient in cholesterol conversion over the remaining cholesterol metabolizing CYPs tested in this study (Fig. 5 and Supplemental Table S4). In all incubations, a single product was found, which was not present in the absence of either the NAD(P)H regenerating system or the redox system. Analysis by GC-MS after TMS-derivatization showed this product has a fragmentation pattern consistent with the TMS-derivative 27-hydroxycholesterol (Supplemental Table S3 and Fig. S4). Previous studies have shown that CYP124A1 and CYP142A1, when reconstituted in vitro with the surrogate spinach redox partners, can further oxidize 27-hydroxycholesterol to the cholestanic acid (Driscoll et al. 2010; Johnston et al. 2010). However, this consecutive oxidation was not observed with *E. coli* or cognate redox partners used in this study. Since cholestanic acid is in the physiological pathway of cholesterol catabolism by Mtb, the results derived from this study suggest that the efficiency or specificity of the cognate redox partners is different from the surrogate spinach redox partners.

**Fig. 6:**
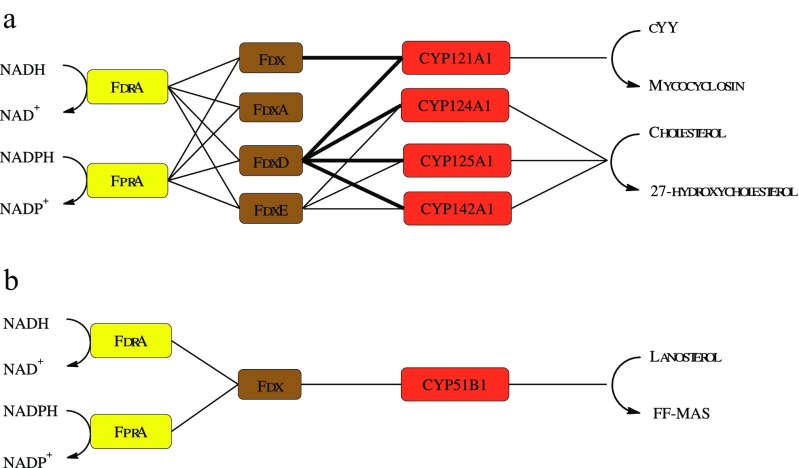
Schematic representation of redox pairs selectivity in Mtb CYPs. **a** Lines indicate identified in vitro specific protein interactions yielding catalytically active Mtb CYPs in this study. **b** In vitro specific protein interactions yielding catalytically active Mtb CYP51B1 identified by Zanno et al. (2005) and McLean et al. (2006). Preferred interactions yielding higher product formation are depicted with darker solid lines. Abbreviations: cYY cyclodipeptide cyclo (L-Tyr-L-Tyr), FF-MAS 14-demethyl-14-dehydrolanosterol

The relative efficiencies for the FNR-Fd combination appeared dependent on the recipient of the electrons, either cytochrome c (Tables 1 and 2) or a CYP (Figs.4 and 5) suggesting that CYP-Fd interactions in Mtb are specific (Fig. 6a).

## Discussion

Mtb genome mapping revealed that it encodes 20 CYPs (Cole et al. 1998). This is a relatively high number of CYPs for a bacterium, since most sequenced bacterial genomes showed only small numbers of CYP genes (Ouellet et al. 2010b). However, the close relative *Mycobacterium smegmatis* contains an even higher number of CYPs, whereas *Mycobacterium leprae* has lost all but one functional CYP protein, encoded by *ML20088*, during adaptation to a host-dependent life cycle (Nelson 2009). The genes encoding several CYPs in Mtb are considered essential due to the roles they play in the viability of Mtb and in the establishment of chronic intracellular infection. In addition, at least two ferredoxins, namely FdxA and FdxC, are also considered essential proteins, highlighting the physiological relevance of the CYP:FNR:Fd system for Mtb viability (Sassetti and Rubin 2003; Xu et al. 2014; DeJesus et al. 2017). In comparison to the 20 CYP genes, *Mtb* codes for only a handful of putative redox-like genes. Therefore, a one-to-one relationship between CYPs and their natural redox partners cannot occur and a more promiscuous electron transfer system should be considered. Several of these have been characterized before (Fischer et al. 2002; Zanno et al. 2005; McLean et al. 2006; Lu et al. 2017), with the exception of FdxA and FdxD. The aims of the present study were to express cognate redox partners and establish for the first time the selectivity in the cognate electron transfer chains for several Mtb CYPs. With the expression and purification of the endogenous proteins, and the reconstitution of physiological Mtb CYP activities, screening of chemical libraries for drug discovery has become feasible.

In this study, we show that FdrA can only accept electrons from NADH, whereas FprA has a preference for NADPH, yielding apparent *K*_*m*_ values of 92 and 1.2 μM, respectively (Supplemental Table S2), in line with previous observations (Fischer et al. 2002; Zanno et al. 2005). The *K*_*m*_^NADPH^ values of FprA are in line with other NADPH-dependent FNRs, such as bovine AdR (*K*_*m*_^NADPH^ = 1.82 μM) and *E. coli* FNR (*K*_*m*_^NADPH^ = 3.9 μM) (Lambeth and Kamin 1976; Leadbeater et al. 2000). Intracellular concentration of NADH/NAD^+^ in Mtb is submillimolar and higher than NADPH/NADP^+^ forms (Kumar et al. 2011). Therefore, the reported low *K*_*m*_^NAD(P)H^ values obtained in this in vitro study in comparison to the physiological NAD(P)H levels in vivo suggest that the cofactor concentrations are unlikely to be the limiting factor for Mtb CYP catalysis. Moreover, the ability of both FNRs to use NADH might be the consequence of an evolutionary adaptation to higher intracellular levels of NADH induced under stress conditions (Rao et al. 2008). Considering that correct Fd-FNR coupling is a prerequisite for efficient electron transfer in the CYP catalytic system, we conclude that NADH-FdrA is the preferred partner for FdxD and FdxE, while Fdx prefers NADPH-FprA (Tables 1 and 2).

Assuming functional genomic clusters, the most likely physiological electron transfer partnerships are Fdx-CYP51B1/CYP123A1/CYP126A1, FdxD-CYP125A1/CYP142A1, and FdxE-CYP143A1/CYP144A1 (Fig. 1). Indeed, CYP51B1 activity has been fully reconstituted in vitro using the cognate redox partners Fdx coupled with either FdrA or FprA (Zanno et al. 2005; McLean et al. 2006) (Fig. 6b). Our results support this theory by presenting the first physiological class I electron transfer chain for FdxD with both CYP125A1 and CYP142A1 (Figs. 5 and 6). However, Fdx and FdxD, but not FdxA or FdxE, can also support CYP121A1 with comparable efficiencies. In addition, FdxE supported in vitro reconstitution of Mtb CYP124A1, CYP125A1, and CYP142A1 catalytic activities, although with lower efficiency (Supplemental Table S4). Unfortunately, substrates for CYP143A1 and CYP144A1, the most obvious CYPs partners for FdxE, have not been identified yet; the same holds true for CYP123A1 and CYP126A1, tentative partners of Fdx. FdxD, the smallest ferredoxin in Mtb with 63 amino acids, appears to be the preferred electron transfer for several Mtb CYPs, while FdxA has yet to be linked to a CYP. Furthermore, the results in this study suggest that the difference in relative efficiencies for the FNR-Fd combination in electron transferring is dependent on the recipient of the electrons, either cytochrome c (Tables 1 and 2) or a CYP (Fig. 4a, 5, and 6a) suggesting that, even though FdxD was able to promiscuously interact with several CYPs in vitro, the CYP-Fd interactions in Mtb are specific.

Productive CYP-Fd interactions have also been reported for other bacterial species. Ewen et al. showed that from the eight myxobacterial ferredoxins, only two (Fdx2 and Fdx8) are able to drive CYP260A1 from *Sorangium cellulosum* So ce56 activity (Ewen et al. 2009). Additionally, Rimal et al. showed that from 56 possible FNR-Fd combinations in *Streptomyces peucetius*, only Fdx1 and Fdx3 are able to support CYP129A2 catalyzed daunorubicin conversion in the presence of FdR2 and Pandey et al. proposed that FprD and FdxH are the major redox partners for CYP105D7 from *Streptomyces avermitilis* (Pandey et al. 2014; Rimal et al. 2015). However, the possible involvement of the non-interactive ferredoxins with other endogenous CYPs was not explored. Here, we studied the ability of natural redox partners to drive several Mtb CYPs and demonstrated the preference of various ferredoxins for multiple CYPs (Fig. 6a). From the endogenous ferredoxins investigated, only the [3Fe-4S] iron-cluster ferredoxins were able to support electron transfer to CYPs. Fdx is shown to cooperate with CYP121A1 (as well as CYP51B1 as shown by others before) (Zanno et al. 2005; McLean et al. 2006). FdxD partners with CYP121A1, CYP124A1, CYP125A1, and CYP142A1 and FdxE with all cholesterol metabolizing CYPs (Fig. 6a). These interactions are a major step towards a complete identification of the physiological electron transfer system in Mtb. Insights into the co-regulation of CYP, FNR, and ferredoxin expression in Mtb, especially during human infection, will contribute to a complete characterization of the interdependence of these proteins. Notably, CYP activity in *S. coelicolor* A3(2) is directly linked to the availability of FNRs during the bacteria life cycle, and FNR expression is induced after exposure to CYP substrates (Lei et al. 2004).

In summary, this study shows the successful expression of two previously uncharacterized Mtb ferredoxins. In addition, Mtb CYPs show specific interactions with more than one ferredoxin (Fdx/FdxD-CYP121A1; FdxD/FdxE-CYP124A1/CYP125A1/CYP142A1), which in turn can operate with two different FNRs, indicating a flexible CYP:FNR:Fd catalytic system in vivo (Fig. 6). This selective promiscuity in the native redox partners might be the outcome of evolutionary advantages of the bacteria to adapt to ever-changing and hostile environments. The present study improves our basic understanding of the selectivity, biochemical properties, and function of the iron-sulfur cluster-containing ferredoxin proteins in Mtb essential for the reconstitution of the cognate CYP:FNR:Fd catalytic system to aid in the development of new antibiotics.

## Electronic supplementary material

ESM 1 (PDF 429 kb)
