# Supplementary material for: Linking cytochrome P450 enzymes from Mycobacterium tuberculosis to their cognate ferredoxin partners
Source: Appl Microbiol Biotechnol. 2018 Aug 22;102(21):9231–42. doi: 10.1007/s00253-018-9299-4 (PMC6208970; doi:10.1007/s00253-018-9299-4)
Supplement: Supplementary file 1 — (PDF 429 kb) [file 253_2018_9299_MOESM1_ESM.pdf]

Applied Microbiology and Biotechnology

**Supplementary Material**

# Linking Cytochrome P450 enzymes from *Mycobacterium tuberculosis* to their cognate ferredoxin partners

Sandra Ortega Ugalde[1], Coen P. de Koning[1], Kerstin Wallraven[2], Ben Bruyneel[3], Nico P.E. Vermeulen[1], Tom N. Grossmann[2], Wilbert Bitter[4], Jan N.M. Commandeur[1] and J. Chris Vos[1*].

[1] AIMMS-Division of Molecular Toxicology, [2] Division of Organic and Peptide Chemistry, [3] Division of Analytical Chemistry and [4] Division of Molecular Microbiology, Faculty of Sciences, Vrije Universiteit, Amsterdam, The Netherlands.

[*]**Correspondence**
J. Chris Vos, Division of Molecular Toxicology, Amsterdam Institute for Molecules Medicines and Systems (AIMMS), Faculty of Sciences, Vrije Universiteit, De Boelelaan 1108, 1081 HZ Amsterdam, The Netherlands.
Tel. +31 205987569
j.c.vos@vu.nl

| **Content** | **Pages** |
|---|---|
| Supplemental Figures | 3-7 |
| Supplemental Tables | 7-13 |
| References | 13 |

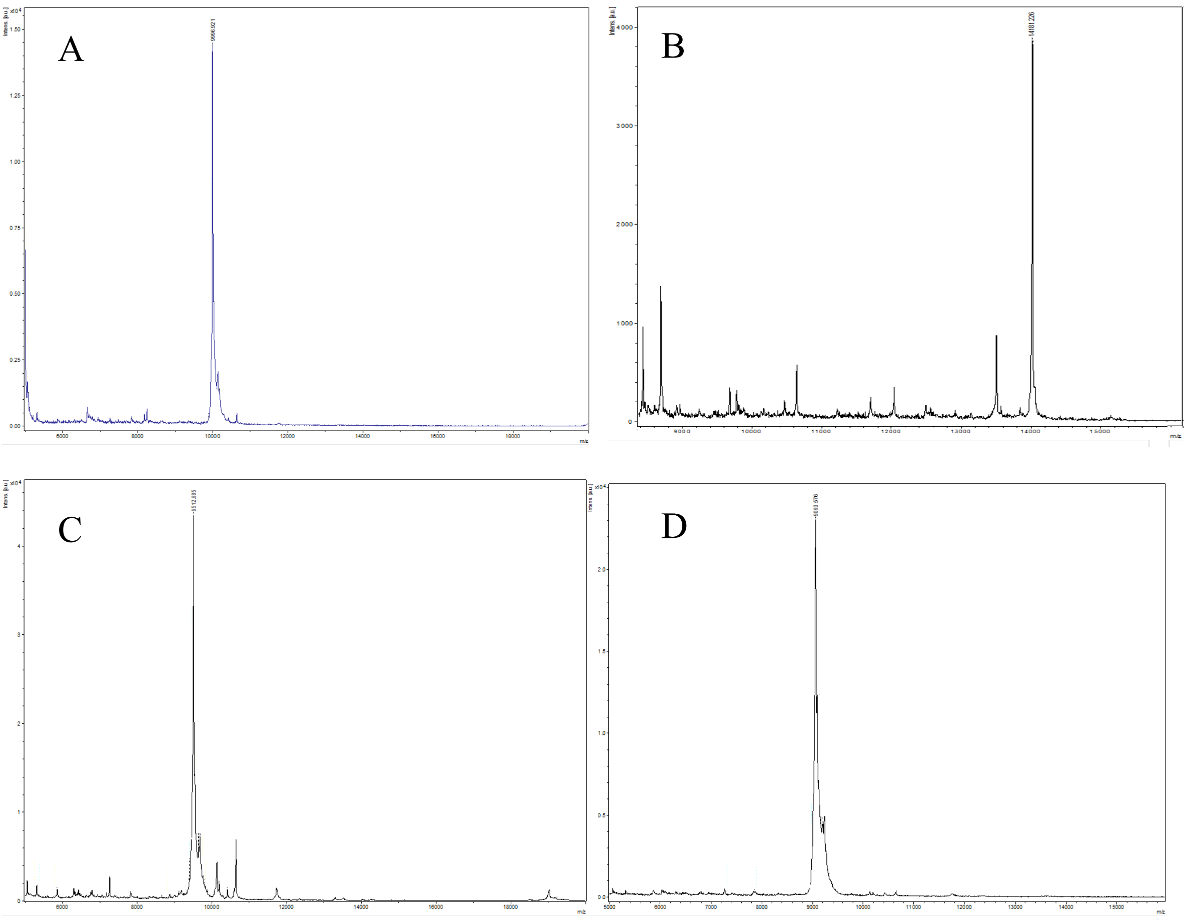


**Fig. S1.** Identification of recombinant Mtb ferredoxins by MALDI-TOF MS analysis. *(A)* Fdx, approximately 10,000 Da. *(B)* FdxA, approximately 14,000 Da. *(C)* FdxD, approximately 9,100 Da. *(D)* Rv1786/FdxE, approximately 9,500 Da was obtained. Samples were analyzed as described in Experimental Procedures.

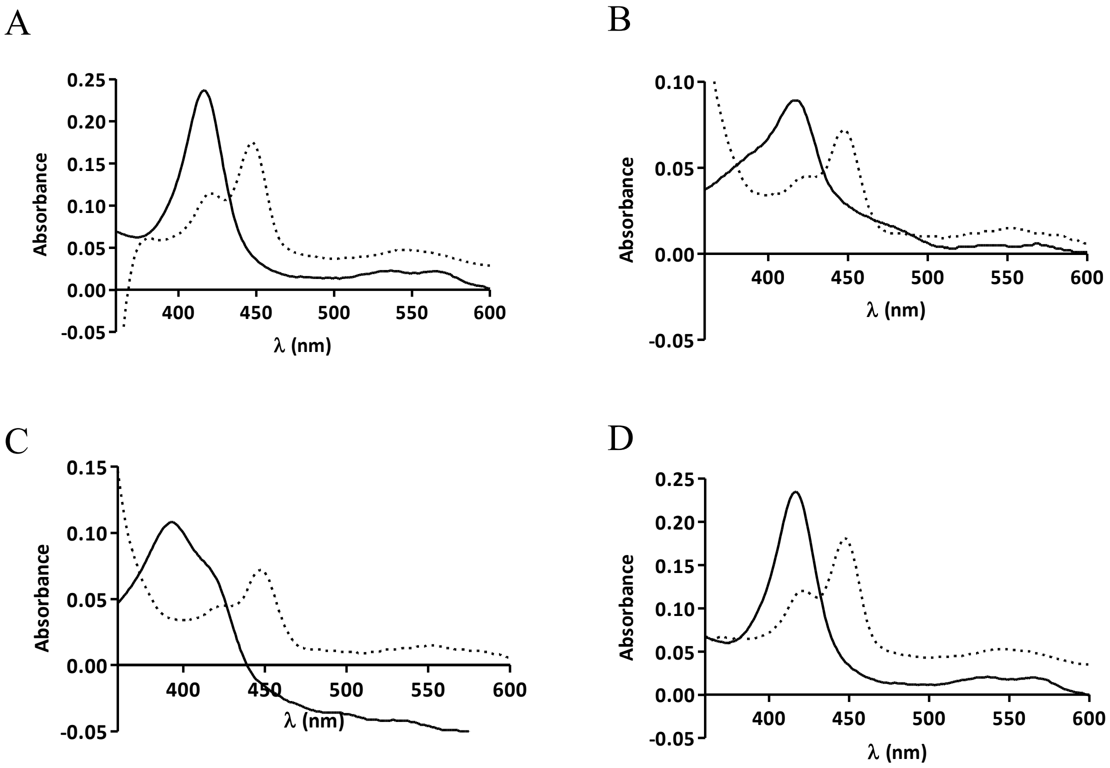


**Fig. S2:** Characterization of recombinant *(A)* CYP121A1, *(B)* CYP124A1, *(C)* CYP125A1 and *(D)* CYP142A1. *Black line*, P450 absorbance spectra in the ferric state; and *dotted line*, ferrous CO-complexed state. Samples were analyzed as described in Experimental Procedures.

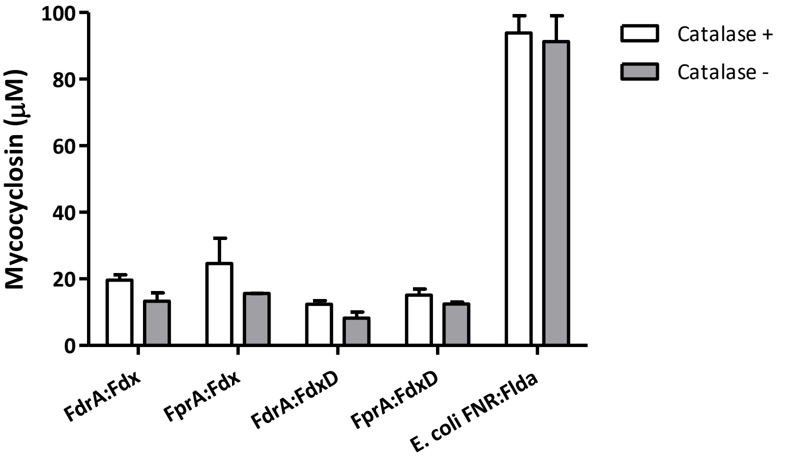


**Fig. S3:** Mycocyclosin formation catalyzed by CYP121A1 (5 µM) supported by cognate redox-partners in the presence or absence of catalase (10 µg/mL)**.** Incubations were conducted in 1:5:10 molar ratio. Error bars represent the variability of duplicates.

**A**

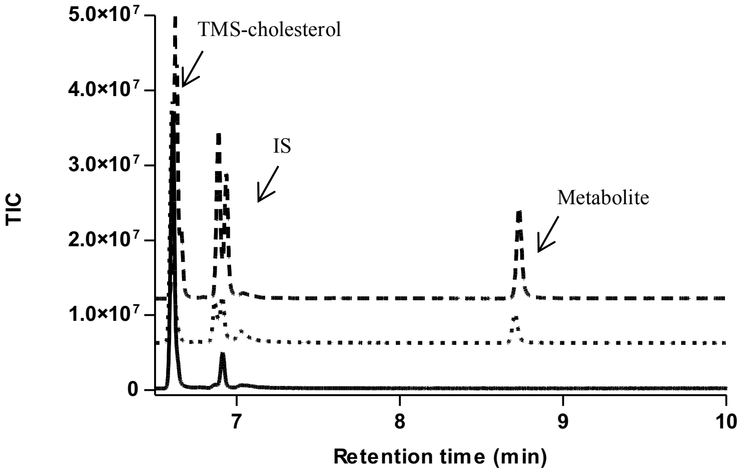


**B**

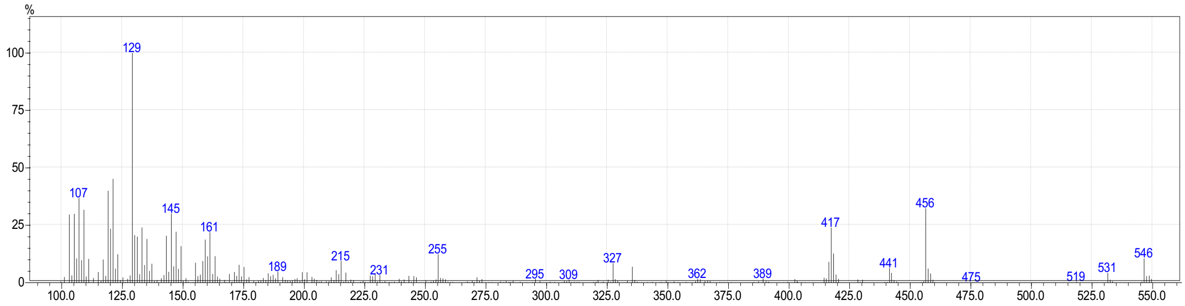


**Fig. S4:** GC-MS separation of the extracts from the CYP142A1 incubations with cholesterol substrate. ***(A)*** GC-MS chromatogram for the incubation of CYP142A1 supported by; *black line;* no CYP142A1; dotted line, CYP142A1:FNR:Flda from *E. coli* in 1:5:10 ratio; and *dashed line*; CYP142A1:FdrA:FdxD from Mtb in 1:5:10 ratio. ***(B)*** Mass spectrum of the major product from the incubation with CYP142A1-FdrA-FdxD catalytic system following GC separation, showing key peaks at *m*/*z* values 417, 456, and 546 that are diagnostic of the methyl ester-TMS ether of the 27-hydroxycholesterol product. Samples were analyzed as described in Experimental Procedures.

**A**

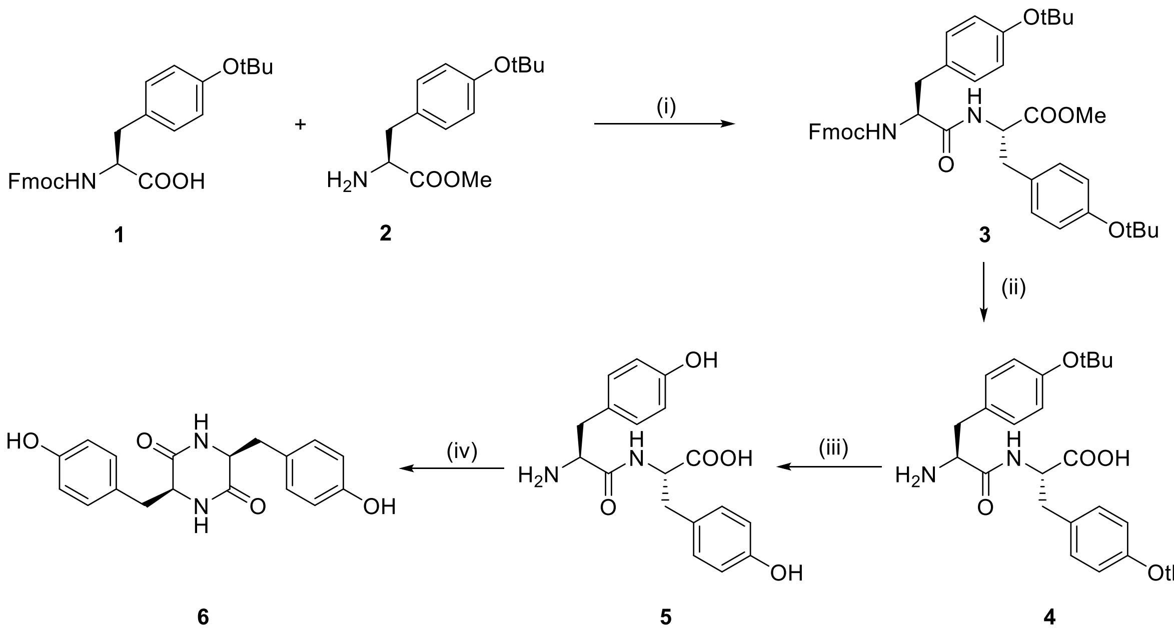


**B**

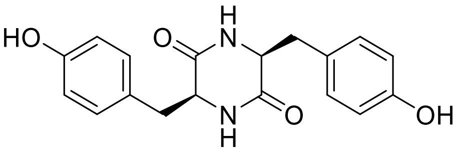


**6**

[1]**H NMR** (500 MHz, DMSO-$d_6$) δ = 6.69 (d, J = 8.2 Hz, 4H), 6.53 (d, J = 8.2 Hz, 4H), 3.74 – 3.68 (m, 2H), 2.36 (d, J = 3.3 Hz, 6H). [13]**C NMR** (126 MHz, DMSO-$d_6$) δ = 166.46, 156.19, 130.89, 126.62, 115.15, 55.82, 38.92.

**Fig. S5:** Schematic overview of cyclo(L-tyr-L-tyr) synthetic pathway. *(A)* Synthesis of cyclo(L-tyr-L-tyr) **(6)**. (i) EDC-HCl, HOBt, DIPEA, DCM, room temperature, 7.25 h; (ii) LiOH, THF:$H_2O$, 0 °C, 0.5 h: (iii) formic acid, room temperature, 2 h; (iv) sec-butanol:toluene, 105 °C, 5 h. The Synthesis of Cyclo(L-tyr-L-tyr) 6 was perfomed according to adapted protocols from Cochrane et al. 2012 *(B)* NMR Data of the final product 6, cyclo(L-tyr-L-tyr).

**Table S1: Sequence alignment of the amino acid sequences of ferredoxins in *Mtb* H37Rv strain genome.** Fdx (*Rv0763c),* 69 aa; FdxA (*Rv2007c*), 114 aa; FdxC (*Rv1177*), 108 aa; FdxD (*Rv3503c*), 63 aa; and Rv1786/FdxE, 67 aa.

| | **Fdx** | **FdxA** | **FdxC** | **FdxD** | **FdxE** |
|---|---|---|---|---|---|
| **Fdx** | 100% | 15% | 17% | 32% | 29% |
| **FdxA** | | 100% | 58% | 21% | 13% |
| **FdxC** | | | 100% | 21% | 13% |
| **FdxD** | | | | 100% | 32% |
| **FdxE** | | | | | 100% |

Abbreviations: aa, amino acid.

**Table S2: Kinetic parameters for the DCPIP reductase reaction of FdrA and FprA.**

| | NADH | | | NADPH | | |
|---|---|---|---|---|---|---|
| | $k_{cat}$ (min$^{-1}$) | $K_m^{DCPIP}$(μM) | $K_m^{NADH}$ (μM) | $k_{cat}$ (min$^{-1}$) | $K_m^{DCPIP}$(μM) | $K_m^{NADPH}$ (μM) |
| **FdrA** | 33 ± 1 | 24 ± 1 | 92 ± 1 | NA | NA | NA |
| **FprA** | 15 ± 1 | 51 ± 1 | 85 ± 1 | 18 ± 1 | 49 ± 1 | 1.2 ± 0.1 |

NA: No activity

**Table S3: Chemical assignments of ions identified in the fragmentation patterns from GC-MS analysis of the major metabolite, 27-hydroxycholesterol**.

| m/z | Description |
|---|---|
| 546 | $M^+$ (molecular ion) |
| 531 | $M^+$ - 15 (loss methyl group) |
| 456 | $M^+$ - 90 (loss TMS group) |
| 441 | $M^+$ - (90 + 15) (loss methyl + TMS group) |
| 417 | $M^+$ - 129 (loss $(CH_3)_3SiOCHCH=CH_2$) (Brooks et al. 1968) |
| 327 | $M^+$ - (129 + 90) (loss 129 fragment + TMS group) |
| 255 | $M^+$ - (ABCD/androstane skeleton, loss TMS group + sidechain (including TMS group)) |
| 129 | 129 fragment, characteristic for TMS-ethers of 3-hydroxyl, $\delta5$ sterols (Brooks et al. 1968) |

**Table S4: Characterization of cholesterol hydroxylation by selected Mtb CYPs supported by surrogate and cognate redox-partners at different molar ratios.**

| TN | | | | |
|---|---|---|---|---|
| **FdrA** | **Molar ratio (CYP:FNR:Fd)** | **CYP124A1** | **CYP125A1** | **CYP142A1** |
| FdrA:Fdx | 1:1:2 | NA | NA | NA |
| | 1:2:5 | NA | NA | NA |
| | 1:5:10 | NA | NA | NA |
| FdrA:FdxA | 1:1:2 | NA | NA | NA |
| | 1:2:5 | NA | NA | NA |
| | 1:5:10 | NA | NA | NA |
| FdrA:FdxD | 1:1:2 | 58.3 ± 1.2 | 33.9 ± 0.9 | 73.7 ± 2 |
| | 1:2:5 | 57.6 ± 0.9 | 26.7 ± 2.7 | 78.7 ± 1.3 |
| | 1:5:10 | 63.7 ± 1.3 | 65.4 ± 2.0 | 81.3 ± 1.5 |
| FdrA:FdxE | 1:1:2 | 1.9 ± 0.2 | 3.7 ± 0.7 | 13.5 ± 2 |
| | 1:2:5 | 2.2 ± 0.6 | 15.1 ± 3.8 | 20.7 ± 2.4 |
| | 1:5:10 | 6.3 ± 1.3 | 15.8 ± 3.1 | 30.3 ± 3.1 |
| **FprA** | **Molar ratio (CYP:FNR:Fd)** | **CYP124A1** | **CYP125A1** | **CYP142A1** |
| FprA:Fdx | 1:1:2 | NA | NA | NA |
| | 1:2:5 | NA | NA | NA |
| | 1:5:10 | NA | NA | NA |
| FprA:FdxA | 1:1:2 | NA | NA | NA |
| | 1:2:5 | NA | NA | NA |
| | 1:5:10 | NA | NA | NA |
| FprA:FdxD | 1:1:2 | 2.2 ± 0.8 | 55.8 ± 0.5 | 68.2 ± 1.3 |
| | 1:2:5 | 13.0 ± 1.5 | 64.8 ± 2.7 | 82.2 ± 0.68 |
| | 1:5:10 | 28.1 ± 0.6 | 62.9 ± 1.3 | 75.65 ± 1.2 |
| FprA:FdxE | 1:1:2 | NA | NA | 7.3 ± 0.7 |
| | 1:2:5 | 3.5 ± 0.1 | 2 ± 1.2 | 8.5 ± 2.1 |
| | 1:5:10 | 3 ± 0.1 | 5.5 ± 0.7 | 5.1 ±1.0 |
| | **Molar ratio (CYP:FNR:Flda)** | **CYP124A1** | **CYP125A1** | **CYP142A1** |
| ***E. coli* FNR/Flda** | 1:1:2 | 25 ± 2.0 | 69.1 ± 1.8 | 75.9 ± 2.5 |
| | 1:2:5 | 47.2 ± 2.1 | 78.4 ± 1.4 | 77 ± 1.9 |
| | 1:5:10 | 61.3 ± 1.7 | 73.7 ± 0.8 | 76.3 ± 3.2 |

TN: Turnover (concentration metabolite over IS (µM)/concentration CYP (µM))

NA: No activity

**Table S5: Oligonucleotide primers used in plasmid construction.**

| Direction | Primer sequence | Restriction site |
|---|---|---|
| F-FprA | 5'- **catatg**cgtccctattacatcgt-3' | *Nde*I |
| R-FprA | 5'- **aagctt**tcagccgagcccaatcc-3' | *Hin*dIII |
| F-FdrA | 5'- **catatg**aacgcacacgtgacca-3' | *Nde*I |
| R-FdrA | 5'- **aagctt**ctaggcctgagtttggtct-3' | *Hin*dIII |
| F-Fdx | 5'- **gctagc**atgggctatcgagtcgaag-3' | *Nhe*I |
| R-Fdx | 5'- **aagctt**ttactctcccgtttctcgg-3' | *Hin*dIII |
| F-FdxA | 5'- **catatg**gtgacctatgtgatcggta-3' | *Nde*I |
| R-FdxA | 5'- **aagctt**ctaagggcactccaccg-3' | *Hin*dIII |
| F-FdxC | 5'- **catatg**gtgacgtacacgatcgcc-3' | *Nde*I |
| R-FdxC | 5'- **aagctt**tcaggcgtcctcgctct-3' | *Hin*dIII |
| F-FdxD | 5'- **catatg**gtgcgggtgatcgtgga-3' | *Nde*I |
| R-FdxD | 5'- **aagctt**ctactcaccacgcgacaa-3' | *Hin*dIII |
| F-Rv1786/FdxE | 5'- **catatg**gtgaaagtccgtctcgatc-3' | *Nde*I |
| R-Rv1786/FdxE | 5'- **aagctt**tcagtccgcgtcgtcct-3' | *Hind*III |
| F-CYP121A1 | 5'- **catatg**accgcgaccgttctg-3' | *Nde*I |
| R-CYP121A1 | 5'- **aagctt**atcctaccagagcaccgg-3 | *Hin*dIII |
| F-CYP124A1 | 5'- **catatg**gggcctcaacacggc-3' | *Nde*I |
| R-CYP124A1 | 5'- **aagctt**tcaggaccacgtaactgg-3' | *Hin*dIII |
| F-CYP125A1 | 5'- **catatg**gtgtcgtggaatcaccagt-3' | *Nde*I |
| R-CYP125A1 | 5'- **tctagat**tagtgagcaaccgggcat-3' | *Xba*I |
| F-CYP142A1 | 5'- **catatg**actgaagctccggac- 3' | *Nde*I |
| R-CYP142A1 | 5'- **aagctt**tcagcccagcggcggg- 3' | *Hin*dIII |

In bold text, the restriction sites are depicted.

## References


Brooks CJ, Horning EC, Young JS (1968) Characterization of sterols by gas chromatography-mass spectrometry of the trimethylsilyl ethers. Lipids 3:391-405. doi: 10.1007/BF02531277

Cochrane JR, White JM, Wille U, Hutton CA (2012) Total Synthesis of Mycocyclosin. Organic Letters 14:2402- 2405. doi: 10.1021/ol300831t
